# Supplementary material for: Splice-Junction-Based Mapping of Alternative Isoforms in the Human Proteome
Source: Cell Rep. Author manuscript; Available in PMC 2020 Jan 15. (PMC6961840; doi:10.1016/j.celrep.2019.11.026)
Supplement: 3 [file NIHMS1546469-supplement-3.zip › DF2/PXD0010154/Prostate-33-O43399-SWHDVQVSSAYKK.pdf]

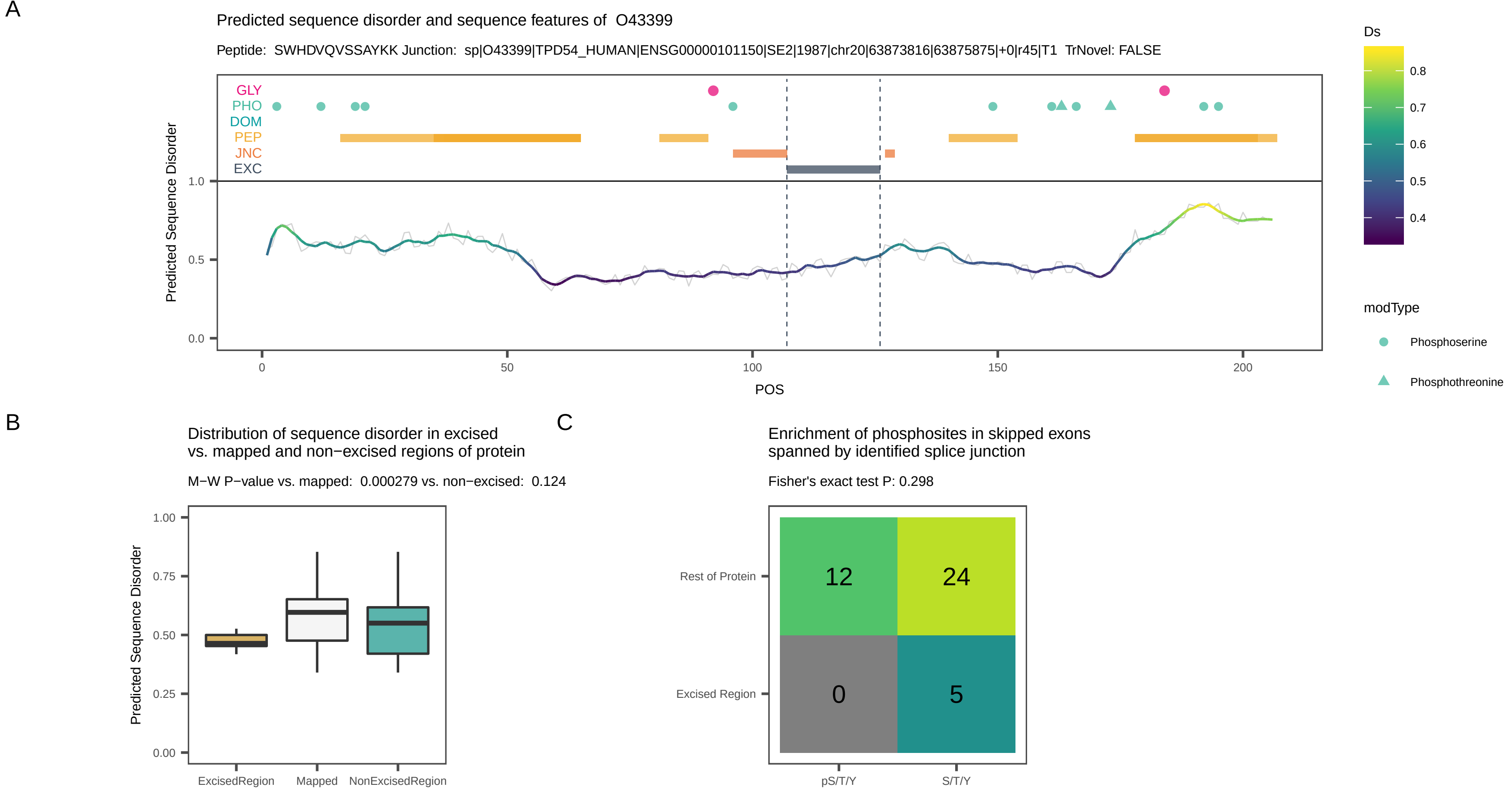
A
Predicted sequence disorder and sequence features of O43399
Peptide: SWHDVQVSSAYKK Junction: sp|O43399|TPD54_HUMAN|ENSG00000101150|SE2|1987|chr20|63873816|63875875|+0|r45|T1 TrNovel: FALSE
GLY
PHO
DOM
PEP
JNC
EXC
Predicted Sequence Disorder
1.0
0.5
0.0
0
50
100
150
200
POS
Ds
0.8
0.7
0.6
0.5
0.4
modType
Phosphoserine
Phosphothreonine
B
Distribution of sequence disorder in excised
vs. mapped and non−excised regions of protein
M−W P−value vs. mapped: 0.000279 vs. non−excised: 0.124
Predicted Sequence Disorder
1.00
0.75
0.50
0.25
0.00
ExcisedRegion
Mapped
NonExcisedRegion
C
Enrichment of phosphosites in skipped exons
spanned by identified splice junction
Fisher's exact test P: 0.298
Rest of Protein
Excised Region
12
24
0
5
pS/T/Y
S/T/Y
